# Pre-Treatment Levels of C-Reactive Protein and Squamous Cell Carcinoma Antigen for Predicting the Aggressiveness of Pharyngolaryngeal Carcinoma

**DOI:** 10.1371/journal.pone.0055327

**Published:** 2013-01-31

**Authors:** Hsuan-Ho Chen, Hung-Ming Wang, Kang-Hsing Fan, Chien-Yu Lin, Tzu-Chen Yen, Chun-Ta Liao, I-How Chen, Chung-Jan Kang, Shiang-Fu Huang

**Affiliations:** 1 Departments of Otolaryngology, Head and Neck Surgery, Chang Gung Memorial Hospital, Linkou, Taiwan, Republic of China; 2 Internal Medicine, Division of Hematology/Oncology, Chang Gung Memorial Hospital, Linkou, Taiwan, Republic of China; 3 Radiation Oncology, Chang Gung Memorial Hospital, Linkou, Taiwan, Republic of China; 4 Nuclear Medicine and Molecular Imaging Center, Chang Gung Memorial Hospital, Linkou, Taiwan, Republic of China; 5 Head and Neck Oncology Group, Chang Gung Memorial Hospital, Linkou, Taiwan, Republic of China; 6 Chang Gung University, Linkou, Taiwan, Republic of China; Shanghai Jiao Tong University School of Medicine, China

## Abstract

The levels of squamous cell carcinoma antigen (SCC-Ag) and C-reactive protein (CRP) can be used to predict tumor invasion, lymph node metastasis, staging and survival in patients with oral cavity cancer. The present study analyzed the relationship between pre-treatment levels of SCC-Ag and CRP in relation to clinicopathological factors in patients with pharyngolaryngeal cancer (PLC) and determined whether elevated levels of CRP and SCC-Ag were associated with tumor metabolic activity via [18F] fluorodeoxyglucose positron emission tomography (FDG-PET). We retrospectively recruited one hundred and six PLC patients between June 2008 and December 2011. All patients received computed tomography (CT)/magnetic resonance imaging (MRI) and FDG-PET staging analyses, and the serum levels of SCC-Ag and CRP in these patients were measured prior to treatment. A SCC-Ag level ≥2.0 ng/ml and a CRP level ≥5.0 mg/L were significantly associated with clinical stage (P<0.001), clinical tumor status (P<0.001), and clinical nodal status (P<0.001). The elevation of both SCC-Ag and CRP levels was correlated with the standardized uptake value (SUV) max of the tumor (≥8.6 mg/L) and lymph nodes (≥5.7 ng/ml) (P = 0.019). The present study demonstrated that the presence of high levels of both pre-treatment SCC-Ag and CRP acts as a predictor of clinical stage, clinical tumor status, and clinical nodal status in patients with PLC. Moreover, elevated levels of SCC-Ag and CRP were associated with a high metabolic rate as well as the proliferative activity measured according to the SUVmax of the tumor and lymph nodes. Therefore, elevated levels of these two factors have the potential to serve as biomarkers for the prediction of tumor aggressiveness in cases of PLC.

## Introduction

Head and neck cancer is the fourth most common cancer and leading cause of cancer-related deaths in Taiwan. [1] Amongst them, pharyngolaryngeal carcinoma (PLC) and oral cancer are prevalent and are associated with adverse lifestyles, including habitual tobacco, areca-quid (AQ) and alcohol use as well as human papilloma virus (HPV) infection. [2], [3].

Knowledge of prognostic factors would be beneficial when evaluating and counseling patients with these cancers. Notably, HPV infection status has been strongly associated with the therapeutic response and survival of oropharyngeal cancer patients; however, it was not shown to be related to tumor stage or clinicopathological factors. [3] Preoperative squamous cell carcinoma antigen (SCC-Ag) level is a marker for pathologic lymph node metastasis, advanced tumor stage, and an increased rate of distant metastasis in patients with oral squamous cell carcinoma (OSCC). [4] Elevated serum CRP, a sensitive marker of inflammation and tissue damage, has been correlated with shorter survival in cancer patients [5], [6], [7], [8] Importantly, the combined use of these two factors is useful in the stratification of OSCC patients receiving radical surgery. [9] However, their significance in patients with pharyngeal and laryngeal cancers has not been carefully addressed.

Fluorodeoxyglucose positron emission tomography (FDG-PET) is a well-established tool for evaluating head and neck cancer. [10] The maximum standardized uptake valve (SUVmax) serves as a semi-quantitative simplified measurement of the tissue deoxyglucose metabolic rate and has been correlated with tumor proliferation rate, tumor grade and the expression of glucose transporters. [11] A high FDG uptake value is generally associated with a less favorable outcome. [12], [13], [14] For example, FDG uptake in breast cancer is correlated with markers of biological aggressiveness that can normally only be evaluated *in vitro* postoperatively, including mitotic count and the Ki-67 labeling index. [15] Accordingly, hypermetabolic breast tumors typically receive a poorer prognosis than those that are hypometabolic, demonstrating the relevance of FDG-PET/computed tomography (CT) analyses to tumor biology. [15].

In this study, we investigated the significance of SCC-Ag and CRP levels in PLC patients and their relationship with various clinicopathological factors as well as 18F-FDG uptake levels on PET scans.

## Patients and Methods

### Patients with Pharyngolaryngeal Cancer

We retrospectively reviewed the charts of all patients newly diagnosed with PLC at our institute between June 2008 and Aug 2011. Patients with distant metastases at diagnosis or who were lost to follow up following diagnosis were excluded. Follow-up commenced at the time of cancer diagnosis and, for this study, completed at the earlier of either December 2011 or death. All patients were evaluated preoperatively with history, examination, routine bloods, chest radiograph, liver ultrasound, FDG-PET and either CT or magnetic resonance imaging (MRI) of the head and neck. [16].

### Treatment of PLC

All patients were staged as per American Joint Committee on Cancer guidelines (AJCC, 2010 edition; [17]) and treated according to their clinicopathological features. As previously described, chemotherapy was administered on an outpatient basis in 14-day cycles and comprised 50 mg/m^2^ cisplatin (P) on Day 1 followed by 800 mg/m^2^ oral tegafur (T) per day and 60 mg oral leucovorin (L) per day for 14 days (PTL regimen). [18] In the chemotherapy/radiotherapy group, chemotherapy was terminated after three cycles if there was little-or-no tumor response. In responders, PTL regimens were continued for up to six cycles before radiotherapy. Patients with good partial responses at the primary site after neoadjuvant chemotherapy received radiotherapy or CCRT for organ preservation.

Radical surgery involved wide excision of primary tumors with at least 1 cm peripheral and deep surgical margins. Patients with advanced tumor stage (T3 or T4), lymph node extra-capsular spread (ECS), tumor depth ≥10 mm or poor tumor differentiation received postoperative radiotherapy or CCRT 4–8 weeks after surgery. [19], [20].

Radiotherapy involved a three-field technique and consisted of conventional bilateral opposing fields with a matching anterior lower neck portal. Daily fractionation size was 1.8 or 2 Gy and five fractions were delivered per week. The planning target volume was created by adding a 5 to 7 mm margin to the clinical target volume. For the group receiving radical surgery first, the post-operative radiotherapy dose was 60–68.4 Gy, depending on the pathology risk factor; for the organ preservation group, the dose range was 68.4–76 Gy.

### Measurement of CRP and SCC-Ag Levels

Pre-treatment serum levels of CRP and SCC-Ag were measured in a fresh blood sample obtained at the time of diagnosis, prior to any form of medical intervention, to minimize inter-individual differences. Serum CRP levels were detected with a high-sensitivity assay (Sekisui Medical Co., Tokyo, Japan) using an auto-analyzer (Hitachi 7600-210; Hitachi Medico, Tokyo, Japan). Serum SCC-Ag level was measured using a commercially available chemiluminescent microparticle immunoassay (Abbott Japan Co., Ltd., Tokyo, Japan). Serum CRP cutoff was set at 5.0 mg/L as this level is internationally agreed to indicate inflammation. [8] The reference cutoff for serum SCC-Ag level was 2.0 ng/mL as previously published. [4], [21].

### FDG PET (PET/CT) Imaging Protocol

FDG-PET/CT was used for the initial tumor survey. Patients fasted for 6 hours before scans and were then subjected to 370 to 444 MBq (10 to 12 mCi), with ^18^F-FDG administered intravenously. An oral contrast agent was administered during uptake time. Next, PET/CT scans (Discovery ST; GE Healthcare) combining a 16-slice spiral CT scanner were performed. The patients were scanned from head to mid-thigh; lower limbs were scanned if indicated. A positive finding was defined as a focus of increased 18F-FDG uptake with intensity higher than that of the surrounding tissues, which was localized according to hybrid images in an area that did not correspond to the physiologic bio-distribution of the radiotracer. Regions of interest (ROIs) were measured for lesions visible on PET images, as well as on simultaneously displayed axial, coronal, and sagittal tomograms. [22] The standardized uptake value (SUV), a semi-quantitative measure of radiotracer uptake, was calculated according to the following formula: SUV = tissue radioactivity concentration [nCi/mL]/[injected dose (mCi)/patient weight (g)]. [23] SUVmax was defined as the highest activity concentration per injected dose per body weight (kg) after correcting for radioactive decay. The SUVmax of the primary tumor (SUVtumor-max) was calculated as the maximum pixel SUV within a ROI encompassing the tumor. The SUVmax of the lymph nodes (SUVnodal-max) was calculated in suspected regions of the neck. The reference cutoff value of the SUVtumor-max was 8.6 and of the SUVnodal-max was 5.7. [22].

### Follow-up

Patients were followed monthly during the first 6 months after treatment, every 2 months for the following 6 months, every 3 months during the second year, and every 6 months thereafter. The patients were subjected postoperatively to hemograms, blood chemistry measurements, chest x-rays, and CT or MRI analyses at the 3^rd^, 6^th^ and 12^th^ month for the first year and then annually for the following 4 years. Patients who presented with abnormal clinical symptoms/signs or laboratory data underwent a bone scan and liver ultrasound for further evaluation.

### Ethics Statement

The study was approved by the Institutional Review Board of Chang Gung Memorial Hospital, Linkou (IRB). The data (CRP and SCC-Ag) were analyzed in retrospective manner. The information was recorded by the investigator in a manner that subjects could not be identified directly or through identifiers linked to the subjects. No informed consent was requested by the IRB.

### Statistical Analysis

The following major variables were examined: CRP levels, SCC-Ag levels, SUV max of the primary tumor and neck lymph nodes, and clinical and pathological lymph node status. The statistical methods used included a univariate analysis with the chi-squared test, and a univariate analysis of survival differences was performed using the log-rank test. A two-sided P value <0.05 was considered statistically significant. These analyses were performed using the Statistical Package for the Social Sciences (SPSS) statistical software, version 19.0 (SPSS, Inc., Chicago, IL, USA).

## Results

### Patient Characteristics

Between June 2008 and August 2011, 106 consecutive patients (103 males; 3 females; mean age 54.3 years, range: 38–96 years) were newly diagnosed with PLC at our institute. The sites of their primary tumors, their clinical staging and the treatments they received are detailed in Table 1. Three patients were excluded from survival analyses as they were lost to follow-up after receiving palliative treatment. Median follow-up period was 14.1 months (range, 0.8–40.8 months). Forty-four patients (41.5%) were disease-free during the follow-up period, 25 patients experienced recurrent disease (23.6%), 17 patients had a partial remission (16%), and 20 patients were stable. Sixteen patients died of their disease during follow-up. The mean serum CRP level prior to treatment was 16.93 mg/L (± standard deviation (SD) 31.68), whereas the mean SCC-Ag level prior to treatment was 2.65 ng/mL (± SD 3.07). All patients also underwent FDG-PET prior to treatment; the mean SUVtumor-max was 13.05 (± SD 6.31), and the mean SUVnodal-max was 8.75 (±SD 6.20).

**Table 1:** Characteristics of the 106 pharyngolaryngeal carcinoma patients.

Characteristic		*n*	%
**Age at onset (years), mean** 54.3 (38–96), **SD** 11.03			
**Gender**			
	Male	103	(97.2)
	Female	3	(2.8)
**Site of primary tumor**			
	Tonsil	29	(27.4)
	Soft palate	17	(16.0)
	Tongue base	12	(11.3)
	Hypopharynx	48	(45.3)
**Clinical Stage**			
	I	4	(3.8)
	II	8	(7.5)
	III	10	(9.4)
	IV	84	(79.2)
**Clinical T-status**			
	T1	14	(13.2)
	T2	24	(22.6)
	T3	15	(14.2)
	T4a	40	(37.7)
	T4b	13	(12.3)
**Clinical N-status**			
	N0	24	(22.6)
	N1	11	(10.4)
	N2a	3	(2.8)
	N2b	32	(30.2)
	N3	24	(22.6)
**Differentiation**			
	Well	6	(5.7)
	Moderate	61	(57.5)
	Poor	31	(29.2)
	Unavailable	8	(7.5)
**Treatment mode**			
	Surgery alone	6	(5.7)
	Surgery with adjuvant radiation	1	(0.9)
	Surgery with adjuvant chemoradiation	5	(4.7)
	Radiation alone	2	(1.9)
	Chemotherapy alone	4	(3.8)
	Concurrent chemoradiation	85	(80.2)
	Palliative treatment	3	(2.8)

* SD: Standard deviation.

### Relationship between CRP level, SCC-Ag level, SUVtumor-max and SUVnodal-max According to Clinicopathological Variables

The cutoff point for measurements of serum CRP level was set at 5.0 mg/L. A close association was observed between a higher CRP level (CRP≥5.0 mg/L) and clinical stage (χ^2^ trend test: P = 0.005), clinical nodal status (χ^2^ trend test: P = 0.003), and tumor differentiation (χ^2^trend test: P = 0.014) (Table 2). A close association was also observed between a higher SCC-Ag level (SCC-Ag ≥2.0 ng/ml) and clinical stage (χ^2^ trend test: P = 0.001), clinical tumor status (χ^2^ trend test: P<0.001), and clinical nodal status (χ^2^ trend test: P = 0.001). Furthermore, higher SCC-Ag levels were often accompanied by higher serum CRP levels (χ^2^ test: P = 0.006).

**Table 2:** The associations between preoperative CRP, SCC-Ag, SUVtumor-max, SUVnodal-max and clinicopathologic parameters (n = 106).

		CRP			SCC Ag		
		[−](n [%])	[+](n [%])	P value*	[−] (n [%])	[+](n [%])	P value*
**Clinical Stage**							
	I (n = 4)	4 (100.0)	0 (0.0)	**0.042**	4 (100.0)	0 (0.0)	**0.005**
	II (n = 8)	6 (75.0)	2 (25.0)	0.005**	7 (87.5)	1 (12.5)	0.001**
	III (n = 10)	7 (70.0)	3 (30.0)		9 (90.0)	1 (10.0)	
	IV (n = 84)	38 (45.2)	46 (54.8)		41 (48.8)	43 (51.2)	
**Clinical T-status**							
	1 (n = 14)	9 (64.3)	5 (35.7)	0.192	12 (85.7)	2 (14.3)	**0.004**
	2 (n = 24)	16 (66.7)	8 (33.3)	0.069**	19 (79.2)	5 (20.8)	<0.001**
	3 (n = 15)	6 (40.0)	9 (60.0)		7 (46.7)	8 (53.3)	
	4 (n = 53)	24 (45.3)	29 (54.7)		23 (43.4)	30 (56.6)	
**Clinical N-status**							
	0 (n = 24)	17 (70.8)	7 (29.2)	**0.011**	19 (79.2)	5 (20.8)	**0.003**
	1 (n = 11)	9 (81.8)	2 (18.2)	0.003**	8 (72.7)	3 (27.3)	0.001**
	2 (n = 59)	25 (42.4)	34 (57.6)		32 (54.2)	27 (45.8)	
	3 (n = 12)	4 (33.3)	8 (66.7)		2 (16.7)	10 (83.3)	
**Differentiation**†							
	Well (n = 6)	5 (83.3)	1 (16.7)	**0.048**	5 (83.3)	1 (16.7)	0.461
	Moderate (n = 61)	37 (60.7)	24 (39.3)	0.014**	35 (57.4)	26 (42.6)	0.511**
	Poor (n = 31)	12 (38.7)	19 (61.3)		18 (58.1)	13 (41.9)	
**CRP**							
	**[−]** (n = 55)				39 (70.9)	16 (29.1)	**0.004**
	[+] (n = 51)				22 (43.1)	29 (56.9)	
**SCCAg**							
	**[−]** (n = 61)	39 (63.9)	22 (36.1)	**0.004**			
	[+] (n = 45)	16 (35.6)	29 (64.4)				
**SUVtumor-max**							
	**[−]** (n = 24)	14 (58.3)	10 (41.7)	0.472	16 (66.7)	8 (33.3)	0.304
	[+] (n = 82)	41 (50.0)	41 (50.0)		45 (54.9)	37 (45.1)	
**SUVnodal-max**							
	**[−]** (n = 42)	28 (66.7)	14 (33.3)	**0.014**	30 (71.4)	12 (28.6)	**0.019**
	[+] (n = 64)	27 (42.2)	37 (57.8)		31 (48.4)	33 (51.6)	

Abbreviation: CRP: C-reactive protein;SCC-Ag: squamous cell carcinoma antigen; SUVtumor-max: maximum standardized uptake valve in tumor; SUVnodal-max: maximum standardized uptake valve in lymph nodes.

CRP (−): CRP level <5.0 mg/L; CRP (+): CRP level ≥5.0 mg/L; SCC-Ag (−): SCC-Ag <2.0 ng/ml; SCC-Ag (+): SCC-Ag ≥2.0 ng/ml;

SUVtumor-max (−): SUVtumor-max level <8.6 mg/L; SUVtumor-max (+): SUVtumor-max level ≥8.6 mg/L; SUVnodal-max (−): SUVnodal-max <5.7 ng/ml; SUVnodal-max (+): SUVnodal-max ≥5.7 ng/ml.

* Chi-square test; **Chi-square trend test; †differentiation: 8 cases the differentiation could not be identified.

When the patients were divided into four groups according to individual pre-treatment levels of SCC-Ag and CRP, a close association was observed between higher levels of SCC-Ag (≥2.0 ng/ml) and CRP (≥5.0 mg/L) and clinical stage (χ^2^ trend test: P<0.001), clinical tumor status (χ^2^ trend test: P<0.001), and clinical nodal status (χ^2^ trend test: P<0.001) (Table 3).

**Table 3:** The associations between preoperative CRP, SCC-Ag and clinicopathologic parameters (N = 106).

		CRP (−), SCC-Ag (−)		CRP (−), SCC-Ag (+)		CRP (+), SCC-Ag (−)		CRP (+), SCC-Ag (+)		
		[n (%)]		[n (%)]		[n (%)]		[n (%)]		P value*
**Clinical Stage**										
	I (n = 4)	4	(100.0)	0	(0.0)	0	(0.0)	0	(0.0)	**<0.001**
	II (n = 8)	6	(75.0)	1	(12.5)	0	(0.0)	1	(12.5)	
	III (n = 10)	6	(60.0)	3	(30.0)	1	(10.0)	0	(0.0)	
	IV (n = 84)	23	(27.4)	15	(17.9)	18	(21.4)	28	(33.3)	
**Clinical T-status**										
	1 (n = 14)	8	(57.1)	4	(28.6)	1	(7.1)	1	(7.1)	**<0.001**
	2 (n = 24)	14	(58.3)	5	(20.8)	2	(8.3)	3	(12.5)	
	3 (n = 15)	3	(20.0)	4	(26.7)	3	(20.0)	5	(33.3)	
	4 (n = 53)	14	(26.4)	9	(17.0)	10	(18.9)	20	(37.7)	
**Clinical N-status**										
	0 (n = 24)	15	(62.5)	4	(16.7)	2	(8.3)	3	(12.5)	**<0.001**
	1 (n = 11)	6	(54.5)	2	(18.2)	3	(27.3)	0	(0.0)	
	2 (n = 59)	17	(48.6)	15	(42.9)	8	(22.9)	19	(54.3)	
	3 (n = 12)	1	(4.2)	1	(4.2)	3	(12.5)	7	(29.2)	
**Differentiation** †										
	Well (n = 6)	5	(83.3)	0	(0.0)	0	(0.0)	1	(16.7)	0.128
	Moderate (n = 61)	26	(42.6)	9	(14.8)	11	(18.0)	15	(24.6)	
	Poor (n = 31)	8	(25.8)	10	(32.3)	4	(12.9)	9	(29.0)	

Abbreviation: CRP: C-reactive protein;SCC-Ag: squamous cell carcinoma antigen; SUVtumor-max: maximum standardized uptake valve in tumor; SUVnodal-max: maximum standardized uptake valve in lymph nodes.

CRP (−): CRP level <5.0 mg/L; CRP (+): CRP level ≥5.0 mg/L; SCC-Ag (−): SCC-Ag <2.0 ng/ml; SCC-Ag (+): SCC-Ag ≥2.0 ng/ml.

* Chi-square trend test;

† differentiation: 8 cases the differentiation could not be identified.

We next compared the association between CRP level, SCC-Ag level, SUVtumor-max and SUVnodal-max and found that the levels of CRP and SCC-Ag were not associated with SUVtumor-max (linear regression, P = 0.070 and P = 0.425, respectively). However, a significant association between CRP level, SCC-Ag level and SUVnodal-max (linear regression, P = 0.023 and P = 0.043, respectively) was observed.

Similar results were also found in the analysis of SUV. When the patients were divided into 4 groups according to pre-treatment SUVtumor-max and SUVnodal-max values obtained by FDG-PET, a close association was observed between higher values of SUVtumor-max (≥8.6) and SUVnodal-max (≥5.7) and clinical stage (χ^2^ trend test: P<0.001), clinical tumor status (χ^2^ trend test: P<0.001), and nodal status (χ^2^ trend test: P<0.001). The associations between the 4 patient groups according to SUVtumor-max and SUVnodal-max and according to the pre-treatment levels of SCC-Ag and CRP were also analyzed and found to be significant (P = 0.019) (χ^2^ trend test: Table 4).

**Table 4:** The associations between preoperative SUVtumor-max/SUVnodal-max and CRP/SCC-Ag (n = 106).

		SUVtumor-max (−)		SUVtumor-max (−)		SUVtumor-max (+)		SUVtumor-max (+)		
		SUVnodal-max (−)		SUVnodal-max (+)		SUVnodal-max (−)		SUVnodal-max (+)		
		[n (%)]		[n (%)]		[n (%)]		[n (%)]		P value*
**CRP and SCC-Ag**										
	CRP (−), SCC-Ag (−) (n = 39)	5	(12.8)	5	(12.8)	16	(41.0)	13	(33.3)	**0.019**
	CRP (−), SCC-Ag (+) (n = 22)	4	(18.2)	2	(9.1)	5	(22.7)	11	(50.0)	
	CRP (+), SCC-Ag (−) (n = 16)	2	(12.5)	2	(12.5)	5	(31.3)	7	(43.8)	
	CRP (+), SCC-Ag (+) (n = 29)	0	(0.0)	4	(13.8)	5	(17.2)	20	(69.0)	

Abbreviation: CRP: C-reactive protein;SCC-Ag: squamous cell carcinoma antigen; SUVtumor-max: maximum standardized uptake valve in tumor; SUVnodal-max: maximum standardized uptake valve in lymph nodes.

CRP (−): CRP level <5.0 mg/L; CRP (+): CRP level ≥5.0 mg/L; SCC-Ag (−): SCC-Ag <2.0 ng/ml; SCC-Ag (+): SCC-Ag ≥2.0 ng/ml.

SUVtumor-max (−): SUVtumor-max level <8.6 mg/L; SUVtumor-max (+): SUVtumor-max level ≥8.6 mg/L; SUVnodal-max (−): SUVnodal-max <5.7 ng/ml; SUVnodal-max (+): SUVnodal-max ≥5.7 ng/ml.

* Chi-square test.

### Combined CRP and SCC-Ag Levels and their Relationships with Prognosis

Regarding the clinicopathological factors influencing patient survival, only tumor status (P = 0.020) and CRP (P = 0.010) had significant influences on overall survival (OS) in the univariate analysis. We combined the CRP and SCC-Ag levels as a variable value for the analysis of prognosis, for which the patients were divided into four groups. For the analysis of all 103 patients in the study, the OS with higher SCC-Ag and higher CRP levels (n = 27) was significantly different to that with non-elevated levels of SCC-Ag and CRP (n = 76) (log-rank test, P = 0.016), although the disease-free survival (DFS) of patients was not significantly different between groups (log-rank test, P = 0.747).

## Discussion

### The Clinical Effects and Molecular Mechanism of SCC-Ag in PLC

The squamous cell carcinoma antigen (SCC-Ag) is a tumor-associated protein that was first isolated from SCC tissues of the uterine cervix. [24] Importantly, this antigen may promote tumorigenesis via a number of different mechanisms. For example, transduction of SCC-Ag into tumor cells has been shown to inhibit apoptosis and promote tumor cell survival, [25] and SCC-Ag can increase cell migration without affecting cell growth, which results in tumor invasion and metastasis. [25], [26] The pro-invasive characteristics of SCC-Ag have also demonstrated positive associations between serum SCC-Ag level and tumor progression, lymph node metastasis and tumor stage. [4], [27], [28], [29], [30], [31], [32] The present study also confirmed the positive relationship between SCC-Ag level and clinical stage, clinical tumor status, and clinical nodal status (P = 0.001, P<0.001, P = 0.001, respectively) (Table 2). In response to stimulation by epidermal growth factor, intracellular SCC-Ag has been shown to translocate to the plasma membrane, and exogenously expressed SCC-Ag then serves to increase cell migration without affecting cell growth. [33] Thus, SCC-Ag may play a role in tumor invasion and metastasis.

### The Clinical Effects and Molecular Mechanism of CRP in PLC

Serum CRP level is a sensitive marker of inflammation that is elevated in response to tissue damage or infection and has been shown to be a prognostic factor in OSCC. [5], [8], [34] This acute-phase reactive protein is produced primarily in the liver, and its expression is up-regulated by pro-inflammatory cytokines such as interleukin-6 (IL-6), interleukin-8 (IL-8) and tumor necrosis factor (TNF). [35] CRP level is elevated in chronic inflammatory environments, which may lead to excessive cell proliferation and subsequent accumulation of DNA damage. The host immune system responds to tumor growth via elevated levels of inflammatory cytokines, which may further increase CRP levels. [36], [37].

Reports from the study of oral cavity SCC have suggested a relationship between CRP level and poor outcomes, pathological tumor status, nodal status and lymph node ECS. [8], [38], [39], [40] However, few studies have investigated the role of CRP in PLC. (Table S1) [38], [41], [42], [43], [44] The current study, which includes the largest number of patients, demonstrated that an elevated CRP level was associated with clinical tumor status (P = 0.005), clinical nodal status (P = 0.003) and differentiation of tumor cells (P = 0.014) in PLC, which is similar to previous findings with OSCC. [8].

### The Correlation between CRP, SCC-Ag and the SUVmax of FDG-PET

FDG-PET has been increasingly applied in head and neck cancer patients for staging and for the study of tumor aggressiveness through the measurement of SUV. [22], [45], [46], [47], [48] Furthermore, studies have demonstrated that the SUVmax values of either the primary tumor or the neck lymph nodes are independent prognostic factors in cases of OSCC. [22], [49] Elevated levels of CRP and SCC-Ag were associated with SUVnodal-max (P = 0.017, P = 0.027, respectively), and the correlation between CRP and SCC-Ag levels were also significant in the current study (P = 0.006).

The combined measurement of SCC-Ag and CRP revealed a more significant correlation with clinical status than either SCC-Ag or CRP when used alone. The patient subgroup with positive levels of both SCC-Ag and CRP demonstrated a profoundly significant association with clinical stage (P<0.001), clinical tumor status (P<0.001) and nodal status (P<0.001) compared to other patient groups (Table 3). The combined value of SCC-Ag and CRP was significantly correlated with the combined value of SUVtumor-max and SUVnodal-max (P = 0.019), which indicates that the preoperative levels of SCC-Ag and CRP together represent a valuable marker for the evaluation of tumor aggressiveness in PLC.

When tumor cells proliferate rapidly, there is an increase in glucose consumption, which be observed as an increase in glucose uptake on a FDG-PET scan. The destruction of nearby tissue and lymph node metastasis is generally accompanied by tissue inflammation, and the infiltration of inflammatory cells and the production of cytokines, especially IL-6, increase the CRP level in the serum. In addition, T-lymphocytes located at the periphery of tumors serve to increase the production of SCC-Ag, [31] which causes accelerated tumor growth to be associated with elevated levels of SCC-Ag and CRP. Moreover, these changes were each reflected as an increase in the SUVmax via PET scan. According to our analyses, the combined use of these two serum markers represents an easy method to evaluate the aggressiveness of PLC tumors. Concerning the FDG-PET analysis, the SUVmax of the primary tumor mass has previously been shown to be related to differences in cellular grade and tumor aggressiveness. [50], [51].

We further analyzed the significance of SCC-Ag and CRP in PLC and found no significant correlation between SCC-Ag level, DFS and OS in a univariate analysis (P = 0.675 and P = 0.313, respectively) (Table 5). In addition, there was significant correlation between CRP level and OS (P = 0.010) but not DFS (P = 0.368) in the univariate analysis(Table 5). The combined value of SCC-Ag and CRP demonstrated a non-significant correlation with DFS (log-rank test, P = 0.747, Table 5) but a significant association with OS (log-rank test, P = 0.016, Table 5). The role of SCC-Ag in human cancers has been demonstrated in a number of clinical studies, [52], [53] which suggests that the serum SCC-Ag level is useful for evaluating responses to radiotherapy and chemotherapy and for predicting early recurrence. This may be related to the molecular role of SCC-Ag in protecting against tumor cell apoptosis. The elevation of CRP arises from the host immune responses to tumor growth with elevated inflammatory cytokines. [36] In patients who received chemo-radiation therapy, CRP is not only an indicator of the host response in the tumor microenvironment but also a reflection of tumor cell killing and local tissue damage [54] either by host or by treatment. From this study, when both SCC-Ag and CRP levels were elevated, the patients could be at risk of worse survival outcome. Further confirmatory studies with a larger number of patients and a longer follow-up period are required due to the limited sample size of the present study. In addition, the radiation responsiveness at different sub-sites should be stratified in a larger number of patients.

**Table 5:** Univariate Log-rank test of prognostic covariates in 103 patients with pharyngolaryngeal squamous cell carcinoma regarding 4-year disease-free and overall survival.

				**Case Number**	**4-year disease-free survival rate (%)**	**P value**	**4-year overall survival rate (%)**	**P value**
**Age (years)**								
		<50		46	40.2	0.308	68.4	0.378
		≥50		57	69.1		56.4	
**Sex**								
		Female		3	100.0	0.259	100.0	0.276
		Male		100	52.6		61.7	
**CRP**								
		<5 mg/ml		54	48.8	0.368	77.2	**0.010**
		<5 mg/ml		49	71.3		40.5	
**SCC-Ag**								
		<2 ng/ml		60	51.2	0.675	64.5	0.313
		≥2 ng/ml		43	60.0		61.2	
**Clinical T-status**								
		T1		14	63.8	0.571	85.1	**0.020**
		T2		24	53.3		85.2	
		T3		14	0.0 (41.4 months)		33.9	
		T4		49	66.2		49.0	
**Clinical N-status**								
		N0		23	68.5	0.069	76.8	0.279
		N1		11	0.0 (41.4 months)		80.0	
		N2		58	56.2		56.1	
		N3		11	100.0 (27 months)		45.0 (27 months)	
**Differentiation** †								
		Well		6	80.0	0.118	66.7	0.806
		Moderate		59	39.8		64.9	
		Poor		30	76.9		59.4	
CRP and SCC-Ag*								
	SCC-Ag (−), CRP (−)			38	49.0	0.747	73.0	**0.016**
	SCC-Ag (−), CRP (+)			16	54.3		87.5	
	SCC-Ag (+), CRP (−)			22	73.4 (37 months)		31.3	
	SCC-Ag (+), CRP (+)			27	68.8		38.5	

Abbreviation: CRP: C-reactive protein;SCC-Ag: squamous cell carcinoma antigen.

CRP (−): CRP level <5.0 mg/L; CRP (+): CRP level ≥5.0 mg/L; SCC-Ag (−): SCC-Ag <2.0 ng/ml; SCC-Ag (+): SCC-Ag ≥2.0 ng/ml.

† differentiation: 8 cases the differentiation could not be identified.

### Conclusions

The present study demonstrated that the combined measurement of SCC-Ag and CRP levels served as a marker of clinical status in PLC and may represent a biomarker capable of predicting prognosis. However, further work is required to elucidate the precise molecular mechanisms for this observed interaction between SCC-Ag and CRP in PLC. As measurements of the levels of SCC-Ag and CRP can be performed quickly, inexpensively and repeatably in a clinical setting, we believe that the levels of SCC-Ag and CRP, as well as the combination of these two factors, could serve as relevant biomarkers of tumor aggressiveness prior to treatment in patients with PLC.

## Supporting Information

Table S1 Literature discussing the role of C-reactive protein (CRP) in oropharyngeal cancer.
(DOCX)
