# Supplementary material for: Pre-Treatment Levels of C-Reactive Protein and Squamous Cell Carcinoma Antigen for Predicting the Aggressiveness of Pharyngolaryngeal Carcinoma
Source: PLoS One. 2013 Jan 31;8(1):e55327. doi: 10.1371/journal.pone.0055327 (PMC3561298; doi:10.1371/journal.pone.0055327)
Supplement: Table S1 — Literature discussing the role of C-reactive protein (CRP) in oropharyngeal cancer. (DOCX) [file pone.0055327.s001.docx]

Table S1. Literature discussing the role of C-reactive protein (CRP) in oropharyngeal cancer.

| Author | Title | Study population | Result |
| --- | --- | --- | --- |
| Gallo O, et al.[38] | Head and neck carcinoma | 18 patients: 9 laryngeal cancer, 4 oral cavity cancer, 3 oropharyngeal cancer and 2 hypopharynx cancer | No analysis on survival. Higher CRP in cancer than normal control patients. |
| Tartour E. et al.[41] | Oropharyngeal cancer | 85 patients : 40 oral cavity cancer, 13 oropharyngeal cancer, 11 hypopharyngeal cancer, and 21 laryngeal cancer | CRP ≥ 10 mg/L related with overall survival (P = 0.003) but not with disease-free survival (P = 0.19). (Follow-up for 24 months) |
| Kulpa J, et al.[42] | Pharyngeal and laryngeal cancer. | 33 patients: pharyngeal and laryngeal cancer. | CRP concentrations significantly higher in T3-4 groups than T1-2 patients. SCC-Ag and/or CRP are independent unfavorable prognostic factors. |
| Dhankhar R, et al.[43] | Head and neck carcinoma | 50 patients: squamous cell carcinoma of the head and neck region | CRP significantly higher in patients with head and neck cancers as compared to the levels in controls (p < 0.001). CRP can help in assessing the severity of disease. |
| Zeng YC, et al.[44] | Advanced laryngeal carcinoma treated with chemoradiotherapy | 57 patients: laryngeal cancer | CRP (> 8 mg/L) was an independent prognostic indicator for cancer specific survival (P = 0.014) |
